# Child and adolescent heart and lung post-transplant adherence

**DOI:** 10.1016/j.jhlto.2025.100293

**Published:** 2025-05-20

**Authors:** Melissa K. Cousino, Jill Plevinsky, Kristin Niel, Emma Rothman, Lauren M. Schnieder, Kelly R. Wolfe, Michael O. Killian

**Affiliations:** a Departments of Pediatrics and Cardiac Surgery, University of Michigan, Ann Arbor, MI; b Department of Psychiatry, University of Pennsylvania Perelman School of Medicine, Philadelphia, PA; c Department of Pediatrics, Emory University School of Medicine, Atlanta, GA; d School of Social Work, Columbia University, New York, NY; e Department of Psychiatry and Behavioral Sciences, Stanford University School of Medicine, Stanford, CA; f Department of Pediatrics, University of Colorado School of Medicine, Aurora, CO; g Florida State University College of Social Work, Tallahassee, FL; h Department of Behavioral Sciences and Social Medicine, Florida State University College of Medicine, Tallahassee, FL

**Keywords:** Pediatric, Transplant, Adherence, Heart, Lung, Thoracic

## Abstract

Although adherence to the prescribed post-transplant regimen is critical to both graft and patient survival following thoracic transplantation, sub-optimal adherence is common among pediatric transplant recipients. Challenges specific to adherence contribute to higher rates of mortality among adolescent and young adult transplant recipients. Multi-directional individual-, family-, community-, system- and society-level factors contribute to one’s self-management skills and treatment adherence. This topical, expert-derived review provides an up-to-date overview of the pediatric thoracic transplant adherence literature with particular attention paid to actionable clinical, research, and policy efforts to enhance treatment adherence among pediatric thoracic transplant recipients, and ultimately, improve graft and quality of life outcomes.

Adherence to the prescribed post-transplant regimen is critical to both graft and patient survival following pediatric heart and lung transplantation. This regimen typically includes daily, time-sensitive medication administration, regular lab and clinic visits, healthy lifestyle behaviors, and infection control precautions. Unfortunately, sub-optimal adherence to these important post-transplant care demands is common among pediatric thoracic transplant recipients. It has been estimated that treatment non-adherence is present in approximately 1 in 3 pediatric transplant recipients, although rates across the solid organ transplant literature range from approximately 4% to 70%.^1^, ^2^, ^3^, ^4^, ^5^, ^6^ Increased risk for non-adherence during the adolescent to young adult (AYA) developmental period contributes to sobering rates of post-transplant mortality.^7^ For example, graft and patient survival is nearly twice as long in adult lung transplant recipients compared to adolescent lung recipients.^8^ Among pediatric heart transplant recipients, more than half of recipients with two or more incidences of reported non-adherence died within two years.^5^

Multi-level, ecological systems theory helps to guide research, policy, assessment, and necessary intervention specific to treatment adherence.^9^, ^10^ Specifically, multi-directional individual-, family-, community-, system- and society-level factors contribute to one’s self-management skills and treatment adherence. Utilizing this framework, this review provides an up-to-date overview of the pediatric post-thoracic transplant adherence literature, with emphasis on studies published in the past decade and/or specific to pediatric thoracic transplant. Established correlates of adherence are reviewed, as well as adherence assessment and intervention considerations. The overall objective of this review is to provide actionable steps for clinical, research, and policy efforts to enhance treatment adherence among pediatric thoracic transplant recipients, and ultimately, improve graft and quality of life outcomes for this patient population.

## Individual-level correlates of adherence in pediatric thoracic transplant

### Patient demographic factors

Patient age has been the most consistently established correlate of adherence, with older adolescent patients demonstrating greater non-adherence and risk for poor transplant outcomes among thoracic transplant recipients, as well as other solid organ groups.^5^, ^11^, ^12^, ^13^, ^14^ Younger children rely entirely on caregivers to manage their treatment, with the responsibility for adherence gradually transitioning to older children and adolescents. Adolescents, on the other hand, face unique challenges as they transition toward greater autonomy. This developmental period is often marked by risk-taking behaviors and prioritizing social activities or peer acceptance over health behaviors.^15^, ^16^ More robust social functioning may help support adherence among adolescent transplant recipients.^17^

The role of gender as a correlate of adherence in pediatric heart transplant patients is less clear,^1^ and few studies have examined gender solely in pediatric thoracic transplant recipients. Of those, no clear relationship between gender and medication non-adherence had been found.^14^ Females solid organ transplant recipients may generally report better adherence than males,^18^ yet greater fluctuation in tacrolimus levels has been observed in females compared to their male peers in both heart-only and multi-organ samples.^1^, ^5^, ^14^ Measurement of adherence may be influenced by social desirability bias or biological differences in tacrolimus metabolism, rather than gender differences in adherence behaviors.^19^ One study suggested that female heart transplant recipients may demonstrate greater non-adherence earlier in the transplant process yet show greater improvement in medication adherence over time when compared to male counterparts.^13^

### Patient mental health

Patient psychological health has long been identified as a key correlate of adherence among pediatric thoracic transplant recipients. Across thoracic only and solid organ samples, pediatric recipients have reported symptoms of depression,^15^, ^20^, ^21^ anxiety,^15^, ^21^, ^22^, ^23^ and posttraumatic stress.^15^, ^24^ Research across pediatric solid organ transplant recipients has shown a consistent link between non-adherence and depression, anxiety, general emotional and behavioral problems, and posttraumatic stress symptoms.^15^, ^17^, ^25^ Non-adherence was associated with depression in a sample of adolescent heart-, lung-, and heart-lung transplant recipients.^26^, ^27^ Trauma and adverse child experiences resulting in child maltreatment and involvement in child welfare services have been linked to poor adherence in pediatric liver recipients.^28^

### Patient neurocognitive functioning

Neurocognitive difficulties, particularly in executive functioning, are common in pediatric heart transplant recipients with preliminary evidence suggesting similar challenges in some pediatric lung transplant recipients.^29^, ^30^ Executive functioning encompasses a constellation of cognitive skills predicting adherence.^31^ Executive functioning difficulties in pediatric thoracic transplant recipients is multifactorial and can vary based upon the presence of an underlying congenital condition, the impact of organ dysfunction to the central nervous system, noxious medical exposures (*e.g.*, lengthy hospitalizations and anesthesia) especially early in life, and potential difficulty with participation in developmentally-stimulating activities, such as school and exercise. The impact of executive dysfunction typically becomes more salient with age as expectations for independence with progressively more complex tasks increases. For adolescent and young adult (AYA) transplant recipients, executive functioning skills play a critical role in readiness for transition to adult care models, experiencing fewer barriers to adherence, and maintaining adherence to the post-transplant regimen.^32^, ^33^, ^34^

## Family-level correlates of adherence

### Family demographic factors

Family factors are also influential in transplant adherence outcomes.^35^ Research in both pediatric heart transplant and across organ groups has consistently found patients from historically minoritized racial or ethnic groups experience greater non-adherence and poorer post-transplant outcomes, highlighting significant health disparities.^5^, ^12^, ^36^, ^37^, ^38^, ^39^, ^40^ Low socioeconomic status (SES) compounds medical adherence challenges. Single-parent households, parents with lower educational attainment, recipients of public assistance, and families with public medical insurance often face additional adherence challenges, as found in samples of pediatric heart and lung transplant recipients.^5^, ^11^, ^41^ Families experiencing significant psychosocial stressors, such as divorce, housing instability, financial concerns, recent death of a family member, or other significant events were associated with significant adherence challenges.^11^ In adult transplant populations, health literacy has been identified as a major factor affecting adherence, and similar challenges are likely present in pediatric heart and lung transplant recipients and caregivers.^42^

### Caregiver mental health

Caregivers may experience significant levels of stress, anxiety, or depression and may struggle to provide consistent support with adherence to the medical regimen.^43^ Parental stress and substance abuse have been found to be associated with medication non-adherence within pediatric solid organ transplant recipients.^35^, ^44^, ^45^ Caregivers experiencing stress, depression, or burnout may feel overwhelmed by the daily demands of parenting while managing a child’s chronic illness, leading to lapses in adherence.^46^

### Family functioning

The interaction between patient and family factors adds complexity to adherence. Families with better adherence have been described as those with greater family cohesion, support, emotional expressiveness, and less internal conflict.^11^, ^17^, ^46^ Increased parental involvement is valuable for promoting adherence, even as the responsibility of adherence from the caregiver to the patient is gradually transitioned.^47^ Families that demonstrate resilience in the face of medical challenges are often better equipped to navigate the complexities of post-transplant care, ensuring better adherence and improved outcomes.^11^, ^35^

## Community, system and society level considerations for adherence

Medication adherence among pediatric thoracic transplant recipients is also influenced by various social and cultural factors. Many families face significant barriers related to SES,^48^ cultural beliefs,^49^ and systemic inequities,^50^ and it is critical to consider the role of community, system, and societal level factors. Children from lower-income households often struggle to access medications, medical visits, and lab draws due to financial constraints, transportation issues, and state sponsored healthcare coverage. In the United States, Black pediatric heart transplant recipients experience higher rates of graft rejection and poorer adherence.^39^, ^40^ It has been suggested, though not thoroughly evaluated, that this may in part be due to structural inequities and provider biases.^5^ While not specific to issues of adherence, studies in Europe and Asia also highlight that immigrant families face challenges in accessing culturally competent transplant care.^51^, ^52^ Other society and cultural level beliefs about health and historical mistrust of medical systems may further influence adherence^53^ in pediatric thoracic transplant; however, there remains a marked paucity of peer-reviewed studies specific to this population.

## Assessment of treatment adherence in pediatric thoracic transplant

Research on the development and validation of standardized assessment of adherence among all pediatric thoracic transplant recipients remains limited despite identification of some patient, family, and society-level correlates of adherence in pediatric thoracic transplantation. The ISHLT 2016 updated listing criteria for heart transplantation,^54^ recent framework for the psychosocial evaluation of pediatric thoracic candidates,^55^ and the earlier works of each Lefkowitz et al.^56^ and Annunziato et al.^57^ call for adherence to be assessed during the pre-transplant psychosocial evaluation. This assessment often centralizes around clinician interview to understand a patient and family’s ability to adhere to various components of an existing treatment regimen, as well as medical chart review specific to adherence. The Pediatric Transplant Rating Instrument (P-TRI), a semi-structured interview for psychosocial evaluation in pediatric transplantation, includes assessment of adherence *via* self-report of the patient and/or caregiver.^58^ Recent revisions, which attempt to improve the initial psychometrics, have been developed,^59^, ^60^, ^61^ but there remains a need for further research to validate a standardized assessment measure.^62^

Post-transplant assessment of adherence to medications (*e.g.*, missed, late, or incorrect doses) *via* self-report questions is a common practice given the feasibility of self-report in a busy clinic environment. The Medical Adherence Measure (MAM), a semi-structured interview designed to assess adherence in pediatric transplant comprehensively assesses various domains and aspects of adherence behaviors. A single-item question, “How many doses of medication have you missed in the past two weeks?” can serve as a screener for adherence concerns. The Adolescent/Parent Medications Barriers Scale (AMBS/PMBS) and the Barriers Assessment Tool (BAT) are often used to understand what barriers, such as forgetfulness, disease frustration, or patient-specific issues, are present and then guide interventions to address the identified barriers.^63^, ^64^

Objective measures, such as automatic pillboxes or Medication Event Monitoring Systems (MEMS) have also been used in pediatric kidney transplant.^65^ Recently, ingestible sensors, which offer objectivity in assessment, have been used across pediatric solid organ groups, but this technology presents unique challenges for consistent implementation.^66^ The Medication Level Variability Index (MLVI) offers an objective biomarker measure of medication adherence by calculating the standard deviation of immunosuppressant levels and can systematically determine those at increased risk of rejection,^67^, ^68^ avoiding bias that can present with clinician ratings of adherence.

## Adherence-focused interventions in pediatric thoracic transplant

The current evidence base for adherence-promotion interventions in youth with chronic medical conditions demonstrates that interventions improve patient-level outcomes, including self-management behavior and disease severity, while also having the potential to improve quality of life, family-level outcomes, and decreased healthcare utilization.^69^ Specific strategies utilized in adherence-focused interventions can be classified as educational, organizational, and/or behavioral.^70^ Educational strategies include diagnosis and related information (*e.g.*, causes of illness), how to manage the illness, and the benefits of adherence. Promoting sustained adherence through patient education is an ongoing process, and other strategies are needed in tandem. Organizational strategies include supporting patient accessibility of the healthcare system, whether it be through streamlining clinic processes or simply fostering improved patient-provider communications and relationships.^70^ For example, clinicians caring for pediatric thoracic recipients may consider involving personnel beyond the pediatric subspecialty medical team, such as primary care.^71^

Behavioral change techniques are defined as observable, replicable, and irreducible components of an intervention designed to alter or redirect a casual process that impacts behavior.^72^ This allows for the ability to tailor interventions for individual patients and their families. Table 1 highlights a sampling of behavioral change techniques to promote adherence. Interventions incorporating behavioral change techniques have shown promise across pediatric chronic illness populations with medium effect sizes.^73^

**Table 1:** Application of Select Behavior Change Techniques to Promote Adherence in Pediatric Heart and Lung Transplant Recipients

Behavior Change Technique	Application
Reward and threat	• Using positive reinforcement by establishing a reward system for consistent adherenc
Antecedents	• Setting up reminder systems such as alarms, phone notifications, or calendar alerts
Associations	• Incorporating medication-taking into the patient and family’s routine (*e.g.*, pairing medication-taking with brushing teeth)
Natural consequences	• Reflecting on natural consequences of nonadherence (see Shaping knowledge)
Feedback and monitoring	• Encouraging use of digital tools to track adherence over time with the opportunity to review for patterns or trends (*e.g.*, poorer adherence on weekends *versus* weekdays)
Goals and planning	• Helping patients set personalized goals for adherence
Social support	• Encouraging the involvement of family members or friends to support and remind patients • Facilitating support groups for patients to share experiences and strategies
Self-belief	• Using motivational interviewing to engage patients in conversations to explore motivations and ambivalence about taking medication
Shaping knowledge	• Providing clear and concise information about the medication, its benefits, and the consequences of nonadherence • Explaining how the medication works and its role in the overall treatment plan

Leveraging technology to support adherence also offers several advantages, namely personalization, access, engagement, and the ability to respond in real-time. Select studies recently published leveraged the personalization capabilities of technology to improve medication adherence in pediatric solid organ transplant recipients utilizing cell phone calls^74^ and text messaging^75^ to improve adherence. Additionally, a recent international review of eHealth interventions across solid organ transplant recipients showed that these interventions may improve adherence up to one-year post-transplant compared to standard care.^76^ eHealth interventions have been piloted with AYA heart transplant recipients with poor adherence and demonstrated improved MLVI and post-transplant outcomes after participation.^68^, ^77^, ^78^

Certainly, understanding the patient-, family- and society-level contributors to treatment non-adherence is of utmost importance in guiding adherence-focused interventions. For example, if depression is resulting in poor sleep and limited motivation for a pediatric thoracic transplant recipient, evidence-based treatment of the mood disorder is of utmost importance for addressing the downstream adherence effects. Similarly, if parental mental health has negatively impacted appointment attendance or supportive monitoring of their child’s medication administration, referral and establishment of parent mental health support are critical to patient adherence outcomes.

## A patient perspective on adherence

“As a heart transplant recipient of thirteen years, I believe that it is critical to redefine adherence or compliance to include building a life worth living. Compliance is not a straightforward journey and the conversation surrounding it needs to acknowledge the gray area that exists once a child leaves the controlled environment of a hospital. The current support model reduces adherence to binary metrics that do not include the lack of control and agency we have over our bodies whenever we take our meds, go to clinic, or miss classes while recovering from biopsies. Both patients and clinicians share the same goals and interests, and I am confident there is an opportunity for clinicians to meet patients where they are in their healing journey to prioritize the patient-clinician alliance. Providing a container where patients feel validated, affirmed, and listened to are interventions that are not cost or labor intensive, and this approach allows the patient to explore their feelings in a safe environment. Prioritizing the relationship clinicians have with their patients is immediately implementable and it will provide a lasting benefit in a patient’s post-transplant journey as they navigate what building a life worth living means to them.”

## Next steps and future directions

Non-adherence to the treatment regimen is a prevalent post-transplant complication, particularly during the high-risk AYA developmental period, for a significant subset of recipients with impact on graft and patient-longevity. Although the pediatric thoracic transplant adherence-focused literature is limited, particularly when compared to pediatric abdominal transplant adherence-related research, some individual, familial, community, and societal-level factors have been identified as important correlates of adherence to the complicated, yet life-sustaining post-transplant regimen. This review underscores critical next steps in terms of advocacy, clinical care, and research for improving adherence among pediatric thoracic transplant recipients.

First, addressing treatment adherence in pediatric heart and lung transplant patients presents important policy and advocacy opportunities. At the policy level, ensuring equitable resources and supports are available for all families, particularly those from marginalized or disadvantaged backgrounds and communities is necessary. Key areas of focus, on a global scale, could include advocacy to promote patient education that is sensitive to variabilities in health literacy, more accessible healthcare systems, and improved data collection to better understand the unique challenges across cultures and geographic regions.

Additionally, advocacy efforts should include increased funding for embedded psychosocial services within pediatric transplant programs, including social work, psychology, child life, and school liaison services. While insurance coverage varies across the world, it is important to underscore that many pediatric transplant recipients do not have adequate coverage for mental health and/or neuropsychological testing services. For those with comorbid mental health concerns or executive functioning deficits, critical adherence-promoting interventions may not be able to be delivered. All patients should have access to psychosocial clinicians with expertise in assessment and behavioral change techniques as part of standardized pre- and post-transplant clinical practice, regardless of referral or insurance coverage. Other barriers to accessing such psychosocial supports and interventions, including time for appointments or identifying a clinician with related expertise, are reduced through these embedded and supported roles.

All members of the multidisciplinary pediatric thoracic transplant team should be responsible for assessing of adherence, not just psychosocial or nursing team members. Training in the use of validating and normalizing statements, as well as motivational interviewing practices, may be fruitful for all team members. Some young people may be most comfortable discussing areas of concern or challenge with a certain provider, regardless of their discipline.

Additionally, while a number of subjective and objective measures of adherence have been developed and published, these are not commonly utilized as part of standard clinical practice due to time barriers, lack of reimbursement or insurance coverage to support use, and limited availability of embedded psychosocial clinicians to address screening results, among others. Finally, it is very important to note that the validity of self-report measures of adherence is limited in low- and middle-income countries (LMICs), as these tools often lack cultural relevance and fail to account for local healthcare challenges. More context-sensitive approaches are needed to assess adherence monitoring globally and within clinical practice.^79^

Despite the important contributions of the research highlighted in this review, significant gaps remain in our understanding of non-adherence in this vulnerable population. Notably, there is a scarcity of large-scale studies focusing specifically on pediatric lung transplant adherence compared to other solid organs. The research is limited by small, single-center studies. National and global pediatric transplant registries include very few data fields specific to adherence and potential contributing factors, such as mental health concerns. This reduces our ability to fully understand prevalence and related factors on a large and global scale.

Moreover, the role of societal and cultural factors in influencing adherence behaviors is insufficiently explored. Structural inequities and biases that potentially impact adherence outcomes, particularly in marginalized and minoritized populations, require thorough investigation. Dedicated research should incorporate diverse demographic groups to better understand and address the unique challenges they face. System-level correlates of adherence have not been well studied. For example, personnel staffing, follow-up care (*e.g.*, number of standardized visits), and patient and family resource supports available at the programmatic and hospital levels likely impact adherence but have not been investigated.

Future research directions should prioritize large, multi-center, longitudinal studies to better characterize adherence behaviors and outcomes over time. These studies should consider adopting standardized adherence assessment tools while utilizing objective metrics like the MLVI to evaluate adherence. Development and validation of technology-driven adherence interventions is needed, such as mobile health applications or use of social media to promote adherence, which have shown promise but remain under-utilized. Importantly, standardized use of these interventions would require hospital- or center-level funding to enable implementation.

Adherence interventions have the capacity to improve adherence behavior among transplant recipients, yet few have been found efficacious in improving transplant outcomes (*e.g.*, biomarkers of organ functioning, rejection, mortality) due to ceiling effects. Those patients struggling the most may experience the most barriers to accessing intervention or participating in clinical trials.^80^ Future adherence-promotion intervention development and implementation requires the deliberate inclusion of patients and families with suboptimal adherence and conscious, patient-centered co-design of interventions to promote engagement and participation.

In summary, addressing non-adherence in pediatric thoracic transplant recipients requires a multifaceted approach, encompassing policy change, clinical practice innovation, and rigorous research. By advocating for equitable resources, embedding psychosocial support within transplant programs, and developing culturally relevant, globally applicable adherence assessments interventions through co-design with patients and families that centralizes on what a life worth living means for them, we can begin to mitigate the complex challenges associated with non-adherence and ultimately improve the long-term health outcomes pediatric thoracic transplant recipients.

## Declaration of Competing Interest

The authors declare that they have no known competing financial interests or personal relationships that could have appeared to influence the work reported in this paper.
